# Production and characterization of melanin pigment from black fungus *Curvularia soli* AS21 ON076460 assisted gamma rays for promising medical uses

**DOI:** 10.1186/s12934-024-02335-y

**Published:** 2024-02-26

**Authors:** Amira S. Abd-EL-Aziz, Nermine N. Abed, Amira Y. Mahfouz, Rasha Mohammad Fathy

**Affiliations:** 1 Botany and Microbiology Department, Faculty of Science, Al-Azhar University (Girls Branch), Cairo, Egypt; 2 Drug Radiation Research Department, Egyptian Atomic Energy Authority, National Center for Radiation Research and Technology (NCRRT), Cairo, Egypt

**Keywords:** *Curvularia soli*, Melanin pigment, Gamma radiation, Antimicrobial, Antioxidant, Anticancer

## Abstract

Owing to the growing need for natural materials in different fields, studying melanin production from biological sources is imperative. In the current study, the extracellular melanin pigment was produced by the fungus *Curvularia soli* AS21 ON076460. The factors that affect the production of melanin were optimized by the Plackett-Burman design (P-BD). The effect of gamma irradiation on melanin productivity was investigated. The maximum melanin yield (3.376 mg/L) was elicited by a stimulus of gamma irradiation at 1.0 kGy. The results evoked that, *Curvularia soli* AS21 ON076460 melanin exhibited excellent antimicrobial activity against all tested bacteria and fungi. *Klebsiella pneumoniae* ATCC 13883 and *P. digitatum* were mostly affected by melanin registering the inhibition zone diameters of 37.51 ± 0.012 and 44.25 ± 0.214 mm, respectively. Moreover, *Curvularia soli* AS21 ON076460 melanin indicated a significant antiviral efficacy (77% inhibition) of Herpes simplex virus (HSV1). The melanin pigment showed antioxidant activities with IC_50_ of 42 ± 0.021 and 17 ± 0.02 µg/mL against DPPH and NO, respectively. Melanin had cytotoxic action against human breast cancer and skin cancer cell lines (Mcf7and A431) as well as exerting a low percentage of cell death against normal skin cell lines (Hfb4). Melanin was effective in wound management of human skin cells by 63.04 ± 1.83% compared with control (68.67 ± 1.10%). The novelty in the study is attributed to the possibility of using gamma rays as a safe method in small economic doses to stimulate melanin production from the fungi that have been isolated. In summary, melanin produced from fungi has significant biological activities that encourage its usage as a supportive medical route.

**Supplementary Information:**

The online version contains supplementary material available at 10.1186/s12934-024-02335-y.

## Background

Melanin is a common, naturally occurring black polymeric pigment occurring in e.g. animals, plants, and microbes [1]. They are produced when phenolic or indolic molecules undergo oxidative polymerization [2]. Melanin is one of nature’s most prevalent, resilient, diverse, and ancient pigments. In the majority of Earth’s living kingdoms, melanin first emerged relatively early [3]. The term ‘melanin’ is derivative from the Greek word melanos which equates to dark. However, additional pigments in this category, such as the pheomelanin present in red hair, warts, and feathers, produce reddish or yellowish colors [4]. Physically, melanin is amorphous, dark brown to black, though red and yellow tints can sometimes be observed occasionally. Melanin controls the color of human skin [5]. Melanin is complex polymer that are resistant to concentrated acids, light, and reducers due to large molecular weight and negatively charged hydrophobic nature. In addition, melanin is heat stable and can withstand thermolysis up to 600º C. On the contrary, it is soluble in alkaline and phenols and is vulnerable to oxidizing agents (bleaching activities) [6].

Microorganisms are more valuable pigment sources than plants and animals because they are easy to produce in low-cost culture conditions with large yields, have no seasonal constraints, and do not compete with genuine crops for scarce agricultural land [7]. Microorganisms produce melanin as a less expensive and more environmentally friendly alternative to chemical approaches [8]. Numerous recent efforts, such as the integration of the statistical approaches and artificial intelligence, the use of economic model substrate, and recombinant microorganisms, are expected to collaborate extensively in future occurrences of microbial melanin production [3]. 1,8-dihydroxynaphtalene (DHN), -glutaminyl-4-hydroxybenzene (HGA), tyrosine, and catechol are used by a variety of fungi to synthesize significant amounts of melanin. Melanin pigments are relatively frequent in fungi, even though melanogenesis is limited to specific growing phases on mycelium, sporulation, or as protective responses against wounds. The melanin precursors in fungi and yeast are secreted and subsequently oxidized exterior of the cell wall [2]. Some *Curvularia* species, which are dematiaceous fungi, are capable of synthesizing melanin which shows a vital function in the virulence and pathogenicity of these fungi [9]. Studying melanin production and the selection of new, atypical sources are critical because of the increased need for the melanin pigment. Microbiological melanin has proven to be useful in a variety of fields. The use of melanin can help a variety of biological, environmental, and technological domains. It has various therapeutic qualities, including anticancer, oxidative stress scavenger, antimicrobial, anti-inflammatory, nanoparticle synthesizer, nerve cell and digestive system protector. Recently, researchers have prioritized research into the prospect of employing melanin as an efficient component in tissue repair engineering [10].

In accordance with the findings of a study performed by Bettinger et al. [11], small melanin films were found to stimulate Schwann neurogenesis in rat pheochromocytoma (PC12 cells) in vitro when compared to collagen films. Fungal melanin inhibits the production of pro-inflammatory cytokines in monocytes, fibroblasts, T lymphocytes, and endothelial cells, giving it immunosuppressive effects. Melanin is utilized as an anticancer therapeutic and to protect radiation patients from the harmful effects of gamma rays during cancer treatment [12].

The current investigation aims to optimize the cultivation conditions for the highest melanin production by the fungus *Curvularia soli* AS21 ON076460, studying the impact of various doses of gamma irradiation on the activation of pigment productivity, and monitoring the antimicrobial, wound healing, cytotoxicity, and antiviral activities of the produced melanin pigment.

## Results and discussion

### Isolation and identification of the isolated fungi

In the current study, a total of ten distinct fungal strains with varying colony morphology were collected from various locations in Egypt. Additional file 1: Table S1 summarizes the morphological characteristics of the isolated fungi under a light microscope.

### Screening of melanin production

**Fig. 1:**
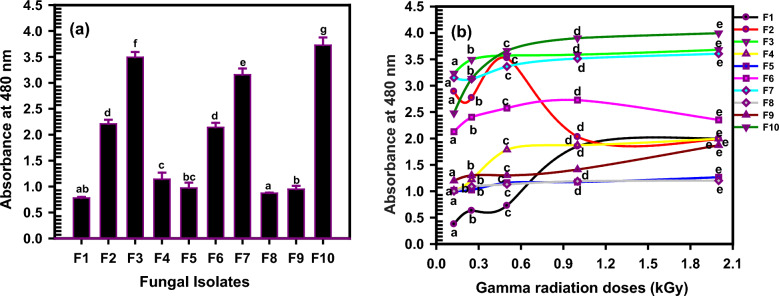
**a** The production of melanin by the isolated fungi. F10 was found to have the superior melanin yield than the other strains. Data are analyzed using one-way analysis of variance (ANOVA) followed by Duncan’s multiple range test that represented by the letters (a,b, c, d). The same letters in represented not significantly different in results at p < 0.05 (**b**) Effect of gamma irradiation doses, 0.25, 0.5, 1.0 and 2.0 kGy on the production of melanin by the isolated fungi.﻿ At 1.0 kGy, melanin was produced sufficiently for all isolates except ﻿F2 that represents the maximum melanin production at 0.5 kGy. The results were plotted as mean values. Error bars represent the SD. According to Duncan’s multiple range test, the same letters represented not significant difference at p < 0.05

The ten different fungal strains were examined to identify the one with a superior melanin yield production. All the isolates were able to produce black-brown culture filtrate. The fungal isolates were cultured on fermentation broth for 7 days and then centrifuged to collect the black melanin pigments. The cell-free supernatant was autoclaved for 20 min in 3 mL of 1.0 M NaOH to extract the melanin pigment. To precipitate the melanin, the alkaline pigment extract was acidified to pH 2 with concentrated hydrochloric acid. The precipitate was rinsed many times with sterilized distilled water, centrifuged for 15 min at 10,000 rpm, dialyzed, and then dried at 60 ºC for 48 h. As shown in Fig. 1a, the different fungal isolates varied in their ability to produce melanin. The results demonstrate that the fungal isolate (F10) was found to have a superior melanin yield than the other strains followed by F3 and F7. The other isolates are less in production and arranged in descending order as follows; F2, F6, F4, F5, F9, F8.

### Consequence of gamma irradiation doses on melanin productivity

The fungal isolates were irradiated at 0.25, 0.5, 1.0 and 2.0 kGy to investigate the influence of gamma rays on their ability in melanin production as in Fig. 1b. The effect of gamma irradiation doses was different on the various isolates. Generally, at 1.0 kGy, melanin was produced sufficiently for all isolates except F2 that represents the maximum melanin production at 0.5 kGy. The fungal isolate (F10) was the best melanin pigment producer at 1.0 kGy.

Gamma irradiation is already used for sterilizing medical devices and drugs such as catheters, drainage bags, and instruments [13]. Extensive relevant research has been performed in recent years about the use of gamma rays for trait development. Gamma rays are also commonly employed as a mutation technique to improve abiotic stress tolerance and disease resistance in crops [14]. Gamma radiation has been widely used because it becomes readily available, cost-effective, and more effective than other ionizing radiations [15].

In our current investigation, the role of gamma irradiation was the improvement of fungal growth and an increase in melanin production. Many researchers interpreted the enhancement activity of gamma irradiation as low linear energy transfer radiation with 1 keV or less that tend to produce clustered non-double strand breaks that can be repaired more resistant than non-clustered DNA harm, as demonstrated using synthetic oligonucleotides. Furthermore, it improves non-DNA-related cell stress through the production of reagents. Gamma radiation has been used effectively to increase cellulase and galactosidase synthesis by microorganisms. *Bacillus subtilis* UTB1 generated a random mutagenesis employing varying levels of gamma irradiation (0.1–3 KGy) to increase its antagonistic action against *Aspergillus flavus* R5 [16]. The effects of gamma radiation on melanotic and nonmelanotic strains of *Cryptococcus neoformans* and *Exophiala dermatitidis* were studied. It was found that, melanin-containing strains developed more quickly than albino strains. Lower radiation doses enhance the development of melanized and nonmelanized *Cryptococcus neoformans*, whereas higher doses improve the growth of melanized cells. The observation that the wild-type (melanotic) strain exhibited ribosome biogenesis gene upregulation due to radiation exposure lead them to speculate that melanin-derived energy was being utilized for protein synthesis [17].

The fungal strain (F10) was identified at the species level using morphological and molecular techniques and considered for further studies. The morphological features evoked the appearance of at least 8.0 cm diameter colonies of the fungal isolate, following 5 days of incubation on PDA at 28 ºC. The colonies often cover the whole Petri dish and show a rapid rate of growth. The mycelia are dense and floccose, mycelium greyish to black, with opaque black reverse. The microscopic characteristics show that the conidiophores are uniform and bent at the points where the conidia originate up to 210 µm length and 8.0 to 10.0 µm width. Conidia size ranged from (8–13) × (19–31) µm, pyriform, brown, multi-septate. The septa (usually 3) are transverse and divide each conidium into four cells. The swelling of the third cell usually gives the conidium a curved shape at an asymmetric cell third from the base attached to the conidiophore.

### Molecular identification of *Curvularia* sp. AS 21 (F10)

**Fig. 2:**
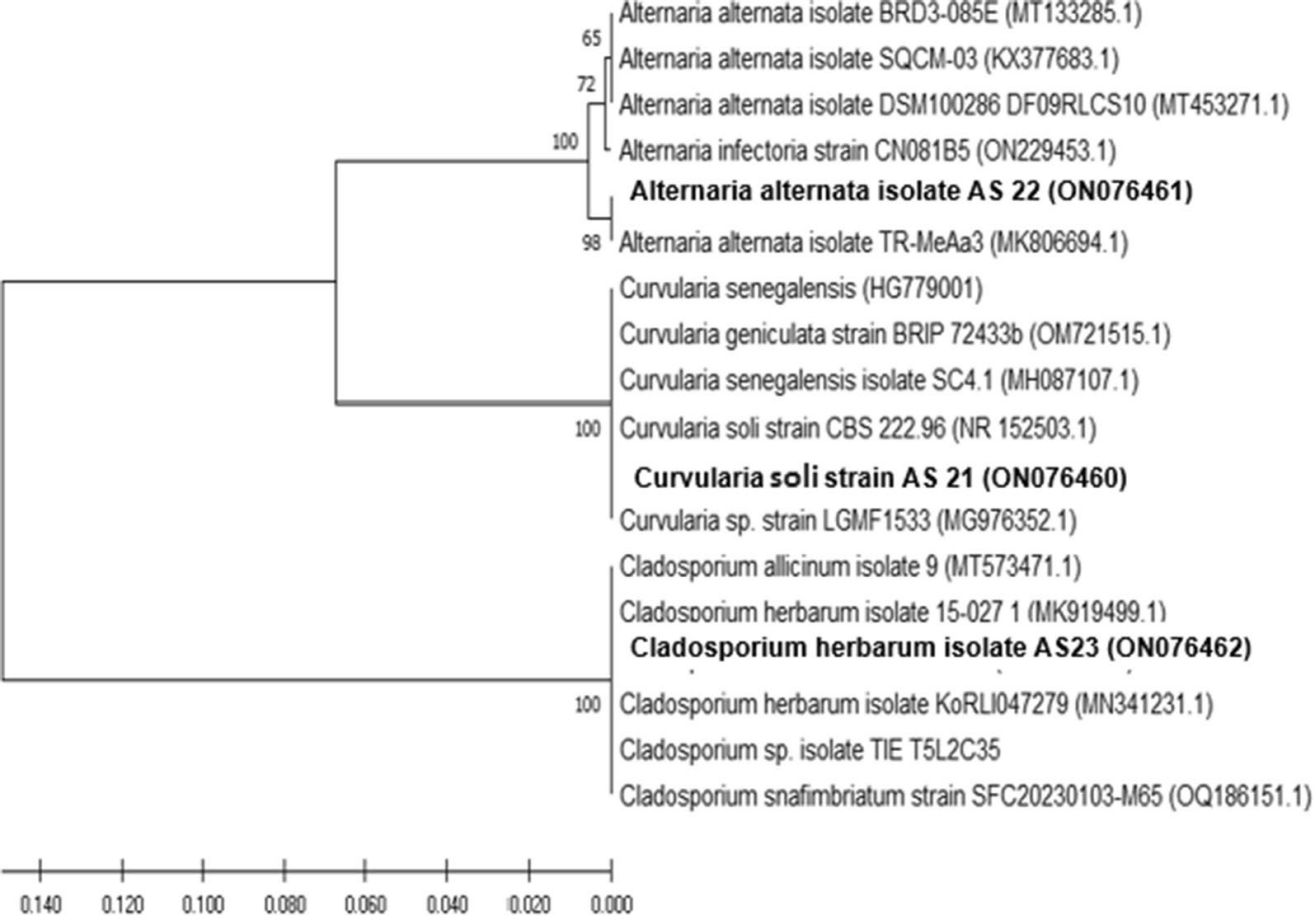
Phylogenetic relationship of *Curvularia* sp*.* AS 21 in a relation with related isolates. The proportion of replicated trees in which the linked taxa and grouped together is exhibited adjacent to the branches. The closest hits of *Curvularia* sp. AS 21 are *Curvularia geniculata* strain BRIP 72433 b and *Curvularia senegalensis* SC4.1

The evolutionary description was concluded utilizing the UPGMA method [18]. The best possible tree with the branch length sum = 20.06473346 was demonstrated. In the bootstrapping test (500 repetitions), the proportion of replicated trees in which the linked taxa are grouped together is exhibited adjacent to the branches [19]. The evolutionary distances were computed using the Maximum Composite Likelihood method [20]. Positions with gaps and missing data were all removed. The final dataset contained 595 locations altogether. Phylogenetic examinations were achieved using MEGA software, version 10.1.6, the dendrogram was created using the utmost likelihood (ML) and neighbor-joining (NJ) method [21]. The closest hits of *Curvularia* sp. AS21 are *Curvularia geniculata* strain BRIP 72433 b and *Curvularia senegalensis* SC4.1. *Curvularia soli* AS21 is the name given to the strain of *Curvularia* sp. (F10) used in this work as a consequence. The achieved *Curvularia soli* sequence was banked in Gene Bank with accession number (ON076460) (Fig. 2). The rRNA sequencing of our *Curvularia soli* AS21 (ON076460) strain is as follows:

1. ACCCCAAGCC GGAAAGTTCG TCAAACTCGG TCATTTAGAG GAAGTAAAAG TCGTAACAAG
2. GTCTCCGTAG GTGAACCTGC GGAGGGATCA TTACACAATA AACATATGAA GGCTGCACCG
3. CCAACAGGCG GCAAGGCTGG AGTATTTTAT TACCCTTGTC TTTTGCGCAC TTGTTGTTTC
4. CTGGGCGGGT TCGCCCGCCT CCAGGACCAC ATGATAAACC TTTTTTATGC AGTTGCAATC
5. AGCGTCAGTA CAACAAATGT AAATCATTTA CAACTTTCAA CAACGGATCT CTTGGTTCTG
6. GCATCGATGA AGAACGCAGC GAAATGCGAT ACGTAGTGTG AATTGCAGAA TTCAGTGAAT
7. CATCGAATCT TTGAACGCAC ATTGCGCCCT TTGGTATTCC AAAGGGCATG CCTGTTCGAG
8. CGTCATTTGT ACCCTCAAGC TTTGCTTGGT GTTGGGCGTT TTTTGTCTTT GGTTTTGTCC
9. AAAGACTCGC CTTAAAACGA TTGGCAGCCG GCCTACTGGT TTCGCAGCGC AGCACATTTT
10. TGCGCTTGCA ATCAGCAAAA GAGGACGGCA CTCCATCAAG ACTCTATATC A

Plackett-Burman design (PB-D) was utilized to optimize the production of melanin by the fungus *Curvularia soli* AS21 ON076460 at gamma irradiation doses of 0.0 and 1.0 kGy. To monitor the impact of one parameter factor on the targeted response, other factors should be constant at a consistent rate. But this procedure does not differentiate between the access findings connected to all examined factors and the overall impact of the parameter factors on the test. The analysis of the 11 variables that may influence melanin synthesis using a Plackett-Burman design was presented in Table 1. The frequency of wavelength 480 nm was used to assess the experimental trial’s results. The absorbance (nm) that represents the generation of melanin was used to characterize the analysis’s results. The high absorbance represents high melanin productivity.

**Table 1:** The responses for the Plackett-Burman design for *Curvularia soli* AS21 ON076460 of the un-irradiated control and gamma irradiated at 1.0 kGy to explore the significant effect of different factors on the melanin productivity

Run	Metals	Carbon source	Nitrogen source	Temp	pH	Incubation/day	Illum	Shaking	Medium type	Tyrosine g/L	NaCl g/L	Absorbance at λ = 480 nm	
												Control	1 KGy
1	Zn	Starch	YE	25	6	21	−	−	DOX	0.5	3	2.865	2.920
2	Zn	Fructose	YE	25	8	15	+	−	PDA	1	3	1.929	2.938
3	Cu	Starch	Peptone	30	6	15	−	−	PDA	1	3	2.688	3.285
4	Zn	Fructose	Peptone	30	8	15	−	+	DOX	0.5	3	2.855	3.065
5	Cu	Fructose	YE	30	6	21	+	+	DOX	1	3	2.732	1.992
6	Zn	Starch	YE	30	8	21	−	+	PDA	1	1.5	2.428	3.327
7	Cu	Fructose	Peptone	25	8	21	−	−	DOX	1	1.5	1.860	2.127
8	Zn	Starch	Peptone	25	6	15	+	+	DOX	1	1.5	3.054	3.325
9	Zn	Fructose	Peptone	30	6	21	+	−	PDA	0.5	1.5	3.042	3.376
10	Cu	Starch	Peptone	25	8	21	+	+	PDA	0.5	3	1.699	2.080
11	Cu	Starch	YE	30	8	15	+	−	DOX	0.5	1.5	2.087	2.128
12	Cu	Fructose	YE	25	6	15	−	+	PDA	0.5	1.5	2.750	3.285

*PDA *potato dextrose agar medium, *DOX*Czapek’s medium, *YE* yeast extract

The results of 12 runs of the irradiated culture (1.0 kGy) show higher melanin production more than the un-irradiated control. Gamma irradiation has an improving effect on the melanin production. The R^2^ value provided a standard from which the test factors could show significant variability in the recorded response values. The R^2^ number is always between 0 and 1. The closer R^2^ to 1, the more efficient the model and is the best to predict the response. The version of Design-Expert 7.0 for ANOVA was used to verify the quadratic equation model, and the outcomes are shown in Additional file 1: Table S2.

**Fig. 3:**
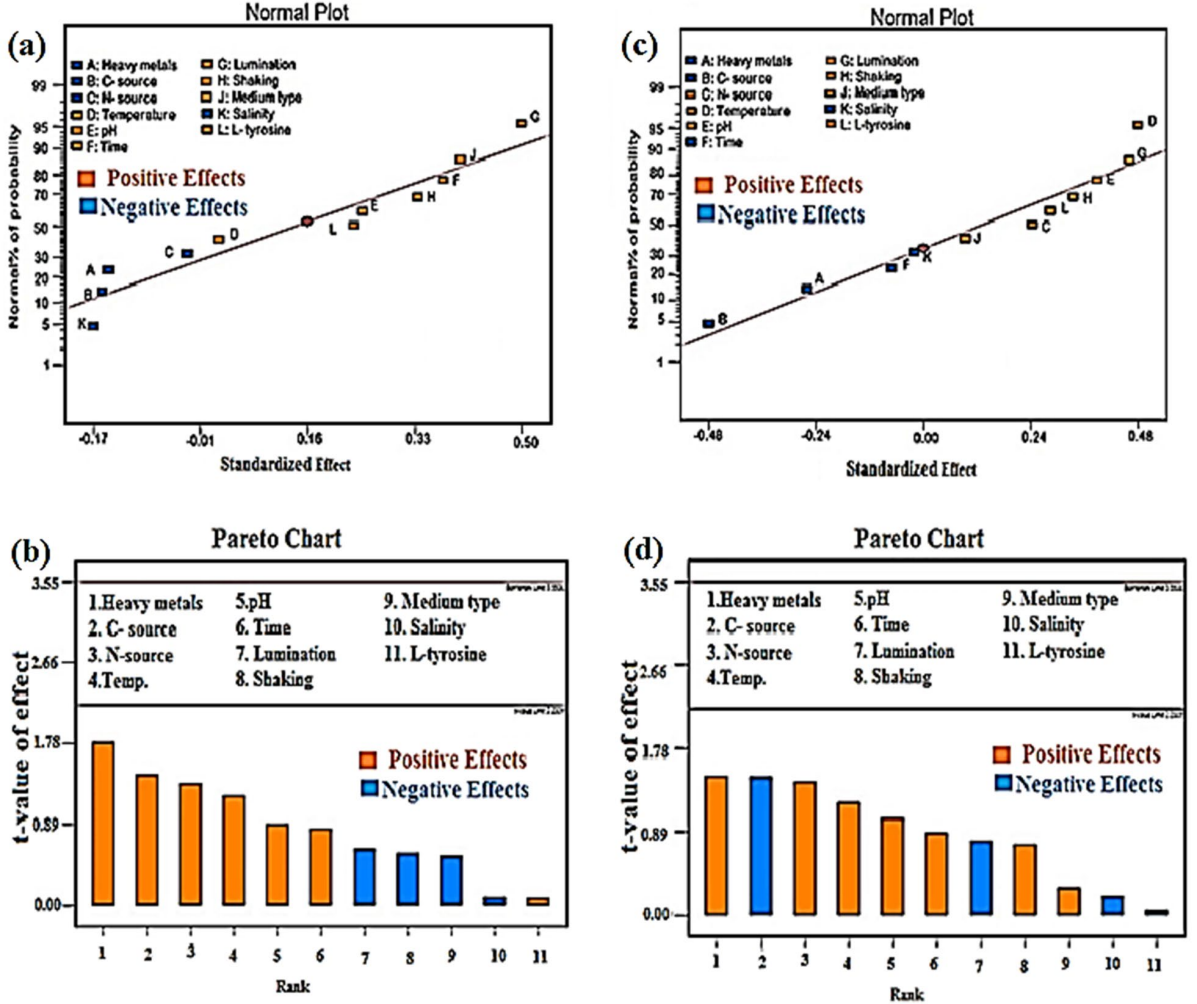
Individual parameter effects on melanin production by *Curvularia soli* AS 21 ON076460 **a** normal plot and **b** Pareto chart for un-irradiated control, **c** normal plot and **d** Pareto chart for 1.0 kGy. The positive effect was represented by orange columns, while the negative effect was represented by blue columns. The extremely optimistic factors on the optimizing conditions for melanin production with the un-irradiated control are heavy metals, C-source, N-source, temperature, pH, incubation time, and L-tyrosine. With gamma irradiation at 1.0 kGy, shaking and medium type have positive effect on the melanin productivity

The statistical analysis listed in Additional file 1: Table S3 and plotted in Fig. 3a, b clarify that the positive factors on the optimizing conditions of melanin production with the un-irradiated control are heavy metals, C-source, N-source, temperature, pH, incubation time, and L- tyrosine. On the other hand, illumination, shaking, medium type, and salinity factors do not have an effect on melanin production (negative effect).

With gamma irradiation at 1.0 kGy, C-source, and L-tyrosine have a negative effect while shaking and medium type have a positive effect on the melanin productivity as represented in Additional file 1: Table S2 and plotted in Fig. 3c, d.

The regression assessments that carried out on the tested records, the test variables, and the variable responses were associated with the subsequent model (Eq. 1) [22] variables of coded factors in un-irradiated control

1 $$= +2.50 A*$$

While the regression assessments equation (Eq. 2) [23] of the irradiation at 1.0 kGy

2 $$= +2.82 A* - 0.13B*- 0.024C*+ 0.12D*+ 0.34E*+ 0.063F*+ 0.20G*= 0.23H*+ 0.17J*- 0.20K*- 0.13L*+ 0.16$$

### Solubility and stability behavior of the melanin pigment

The dried extracted melanin pigment produced by *Curvularia soli* AS21 ON076460 was examined for the physicochemical tests (Additional file 1: Table S4). The melanin pigment was tested for its solubility in different solvents. The results evoked its solubility in 0.1 mol/L NaOH, and 0.1 mol/L KOH, but insoluble in water, chloroform, ethanol, and acetone. As shown in Additional file 1: Table S4, the decolorization of the extracted melanin pigment by the oxidant agent hydrogen peroxide (H_2_O_2_) was analyzed. The melanin pigment was decolorized by hydrogen peroxide.

When the extracted melanin was precipitated for polyphenols with FeCl_3_ and 3.0 M hydrochloric acid (HCl) it appeared as a dark brown. All these properties are similar to those noticed in the ordinary melanin pigment (Additional file 1: Table S4).

**Fig. 4:**
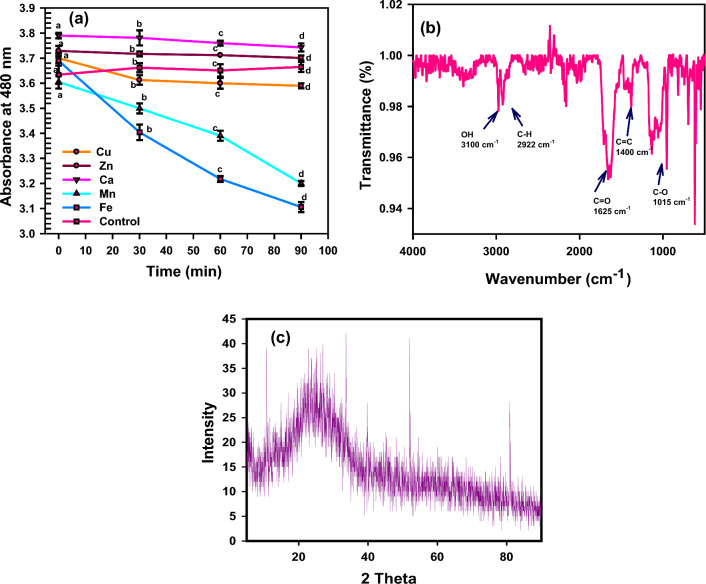
**a** Effect of metals on the stability of melanin, Ca and Zn have positive effect on melanin production. Fe and Mn decreases melanin production produced by *Curvularia soli* AS21 ON076460 compared with control. The results were plotted as mean values. Error bars represent the SD. According to Duncan’s multiple range test, the same letters represented not significant difference at p < 0.0﻿5 (**b**) Fourier-transform infrared (FTIR) spectrum showing the functional groups of melanin produced by *Curvularia soli* AS21 ON076460. **c** XRD spectrum of the melanin sample

The granules of melanin generated are nanoaggregates of oligomers including various monomers. Although melanin’s primary biological function is to defend against sun radiation damage, it may also be involved in the defense against oxidative stress. The effect of metals on the stability of melanin was studied. It was found that Ca^2+^ and Zn^2+^ increase the absorbance of melanin at λ = 480 nm. While Mn^2+^ and Fe^2+^ reduce the absorbance. Cu^2+^ keeps the stability of melanin (Fig. 4a). Interestingly, several studies have discussed similar findings in which the combination of metal ions with melanin could influence the outcome of melanogenesis. Cu(II) ions induce the rearrangement of dopachrome to DHICA. Zn(II), Ca(II), and Mg-(II) bind to COOH groups. Concerning the entire ability of melanosomes to interact with individual metals, it is essential to consider that different binding sites might be connected with other functional groups, such as NH, COOH, and OH. It is probable that the same metal is coordinated by more than one site. The capability of melanin to act as a reservoir for metal ions, allowing, exchange, release, and storage, as well as firmly bind and bind reactive metals, may decrease their function in producing oxidative stress [24]. Melanins, both synthetic and extracted, have unique features such as electrical conductivity and a strong propensity for attaching to molecules, including heavy metals [25].

The natural melanin disrupts in the presence of Fe ions, and amorphous organization predominates with Cu and Zn ions. Unfortunately, when natural melanin interacts with metals, it becomes a colloidal solution. The conductive phase produced by dispersed metal ions in melanin solution might clarify the improvement in melanin solutions conductivity doped with metals such as Zn, Fe, and Cu. The increased melanin-metal ions conductivity could be attributed to induction in conduction routes number formed between the particles aggregated in the colloid [26].

### Tyrosinase enzyme activity assay

The dopachrome production by *Curvularia soli* AS21 ON076460 control (without irradiation) and irradiated at 1.0 kGy was evaluated spectrophotometrically at λ = 475 nm. As indicated from the results, the dopachrome formation reveals tyrosinase activity of 411 ± 0.476 and 477 ± 1.32 U/mL for control and irradiated, respectively. Tyrosinase activity was found to be slightly greater in the irradiated sample rather than in the control.

Melanin pigments are negatively charged, hydrophobic, and have high molecular weight. Melanin is generated by the oxidized polymerization of phenolic and indolic substances. Melanin is produced by fungi in two pathways; the l 3–4 dihydroxyphenylalanine (l-DOPA), or the 1,8-dihydroxynaphthalene (DHN). The l-DOPA pathway is activated by tyrosinase and laccase, wherein tyrosine is transformed to l -DOPA, formerly to dopaquinone. In the DHN pathway, malonyl-CoA is produced internally, and then activated with polyketide synthases. The successive decarboxylative condensing of a total of five malonyl-CoA molecules yields 1,3,6,8-tetrahydroxynaphthalene, and it is then exposed to reduction and dehydration processes, yielding 1,8-dihydroxynaphthalene (DHN). DHN polymerized to DHN melanin [27, 28]. The melanin of *Curvularia soli* AS21 ON076460 is biosynthesized from tyrosine.

### Characterization of melanin pigment

FTIR analysis can be used as a quantitative approach to investigate the bonding interactions in substances. Because molecular vibrations are closely linked to molecule symmetry, it is often easy to deduce the way a molecule bonds to surfaces or acts as part of the infrared analysis [29]. The FTIR analysis of the extracted melanin represented in Fig. 4b indicates the distinctive absorption bands and their wavelength values identical to the typical melanin precursors. The FTIR analysis of melanin is in the area of 3300 cm^−1^ to 500 cm^−1^. The observed peaks of melanin are 3100 cm^−1^, 2922 cm^−1^, 1625 cm^−1^, 1400 cm^−1^to 1500 cm^−1^, and 1015 cm^−1^. FTIR assay was similar to the outcomes reported by the previous investigators [30, 31]. FT-IR analysis reveals extending bands at OH phenolic groups in the 3100 cm^−1^ range. Moreover, the band at 2922 cm^−1^ is assigned to stretching vibrations of the aliphatic C–H groups. The band at 1625 cm^−1^ is assigned to vibrations of aromatic ring C=O groups, while bands from 1400 to 1500 cm^−1^ may be instigated by the aliphatic C=C group. The band at 1015 cm^−1^ is assigned to the C-O group. Finally, identical peaks in the area of 3300 cm^−1^ to 500 cm^−1^ were observed during FTIR analysis, and these peaks were correlated to those found in other melanin-producing species and a melanin standard. These characteristic absorption peaks of the melanin extracted from *Curvularia* sp. AS21 ON076460 are similar to those observed for DOPA, and DHN melanins FT-IR reference spectrum reported by Mbonyiryivuze et al. [32] except some peaks where they reported the absorption peaks at 2917 cm^−1^ and 2839 cm^−1^ to the stretching vibration of the aliphatic C–H group. The characteristic band at 1621 cm^−1^ is attributed to the vibrations of the aromatic ring C=C and C=N bond of the aromatic system in addition to the C=O double bond (COOH) of the carboxylic function. The peaks between 1468–1330 cm^−1^ can be due to aliphatic C–H groups. The weak bands below 700 cm^−1^ are ascribed to alkene C–H. The OH bending of the phenolic and carboxylic groups was at the peak of 1374 cm^−1^. The peak at 1038 cm^−1^ is an indication of the CH group.

The X-ray diffraction spectrum of the extracted melanin is distinguished and characterized by a broad peak that is commonly observed in amorphous and disordered spectra. As presented in Fig. 4c, the reflections of 2θ for the diffraction peaks have been identified at 10.69, 22.42, 33.65, and 52.89, respectively. The characteristic peaks of the melanin pigment were indicated in previous studies. The XRD pattern of melanin characterized by a broad peak shows that it lacks crystalline structure [33]. In line with the current study, many investigations have reported that the natural melanin granules that are extracted from fungi are amorphous and range in size from 30 to 1000 nm, depending upon the extraction source [17, 34].

**Fig. 5:**
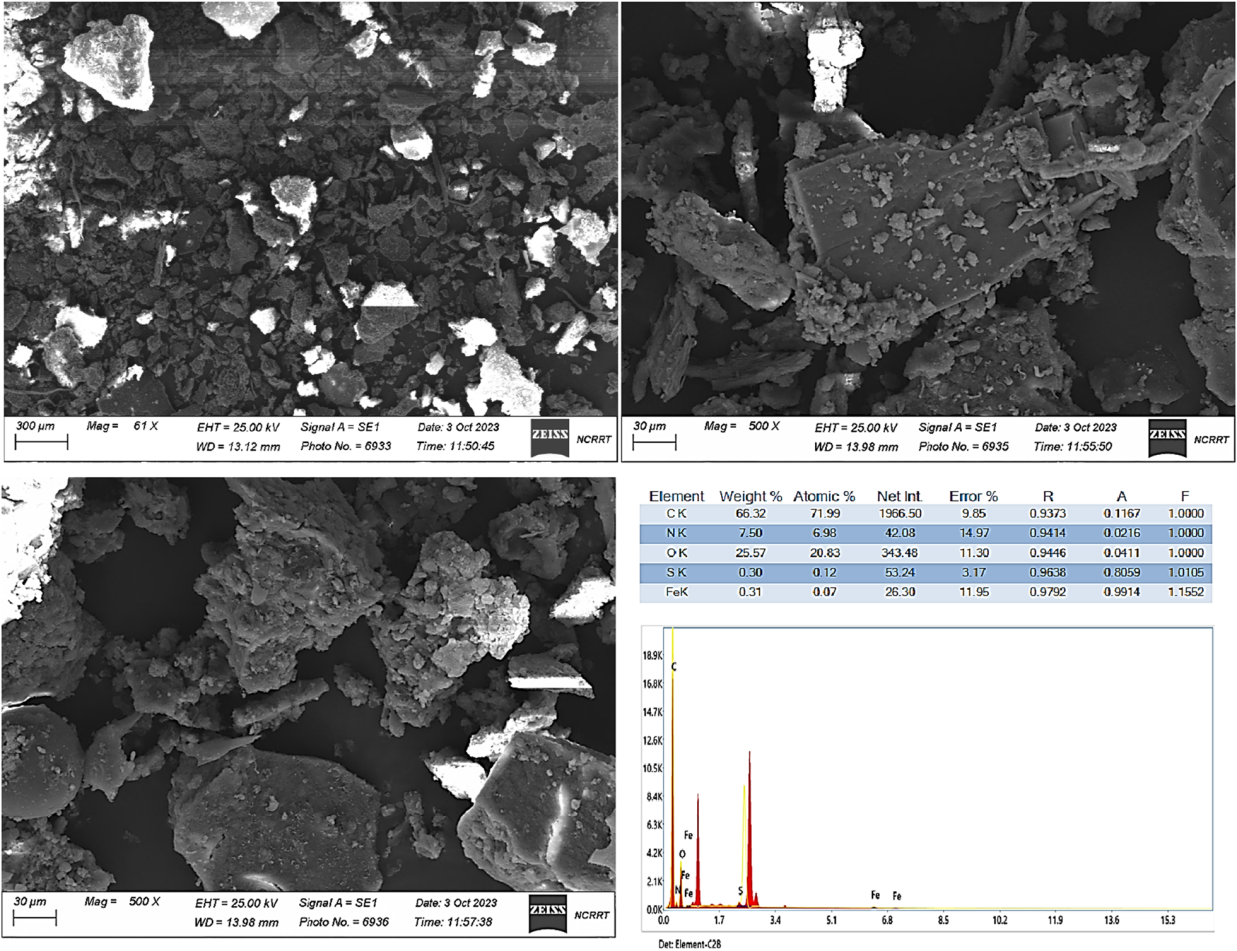
SEM analysis of melanin particles produced by *Curvularia soli* AS21 ON076460 at different magnifications, 1000 and 500 X shown that melanin has average size 130–160 nm. SEM films revealed that crude melanin was free of cells and mycelium. EDX analysis revealed the elements constituents of melanin with their weight and atomic %. The most abundant elements are carbon and oxygen with weights of 66.32 and 25.57%, respectively

SEM analysis of the morphological characteristics and structural organization of the extracted melanin reveals that the melanin of *Curvularia soli* AS21 ON076460 has distinct particles (Fig. 5). The average size of the extracted melanin particles is 130–160 nm. Moreover, SEM films reveal that crude melanin was free of cells and mycelium. As a result, that would be an advantage if the melanin were considered for use in medical therapies.

The EDX analysis is mainly reliant on the interface of X-ray excitation on the studied specimen. The results reveal that the most abundant elements had outstanding proportions for atoms of carbon and oxygen, with weights of 66.32 and 25.57% and atomic percent’s of 71.99 and 20.83%, respectively. Nitrogen and sulfur atoms are present in less weight of 7.5 and 0.3% with atomic % of 6.98 and 0.12. Compared with the sulfur content of eumelanin (0.09%) and phaeomelanin (9.78%) [35], the content of sulfur in *Curvularia soli* AS21 ON076460 melanin is 0.3%, which is higher than that of eumelanin. Moreover, a former investigation [36] displayed that the nitrogen content in 5,6-dihydroxyindole eumelanin is 6 to 9%, which was consistent with our study that registered 7.5% of nitrogen content. These results indicated that *Curvularia soli* AS21 ON076460 melanin could be classified as eumelanin.

### The bioassays of the melanin pigment

#### The antibacterial activity

**Fig. 6:**
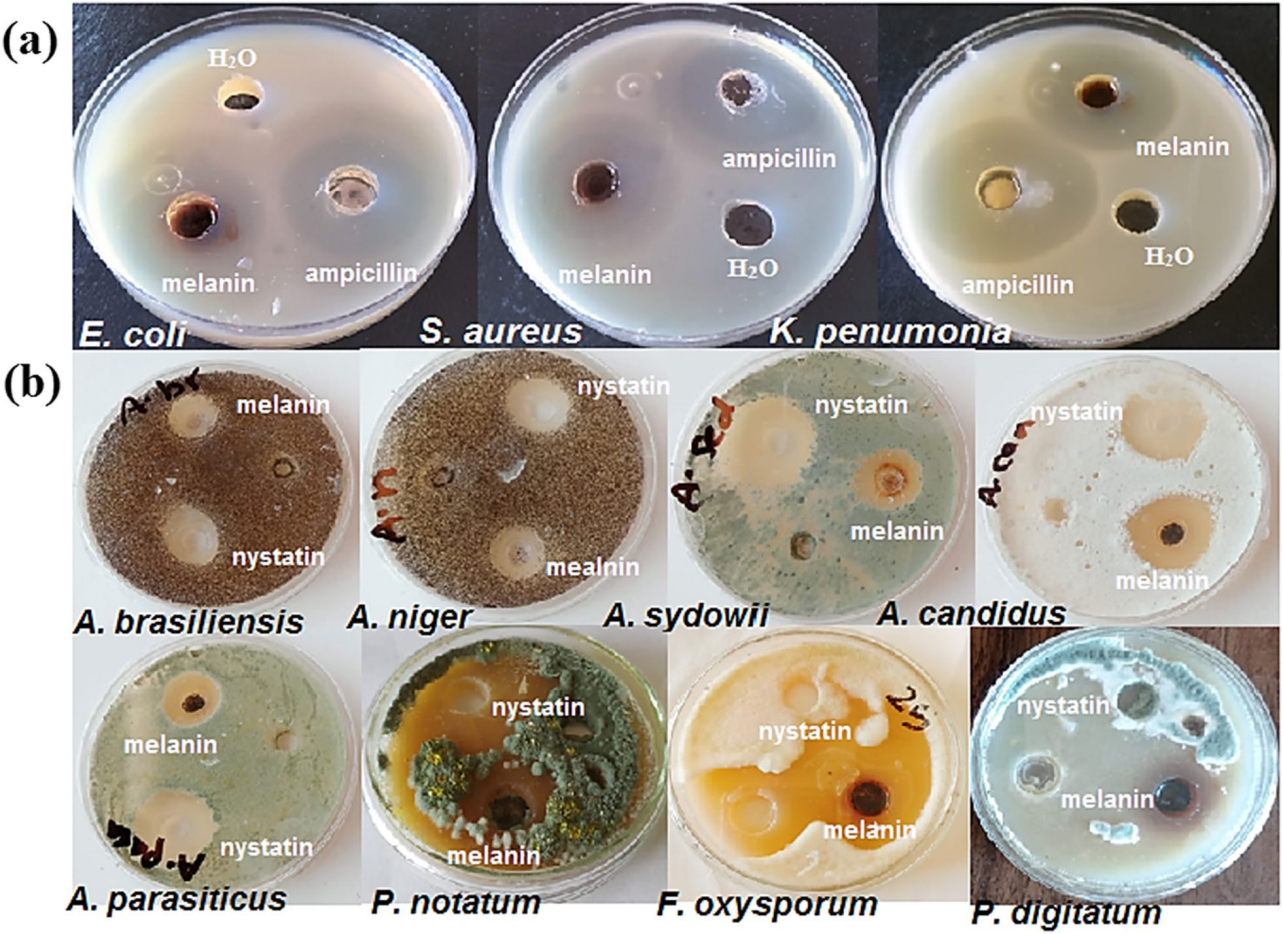
**a** The antibacterial activity of melanin pigment produced by *Curvularia soli* AS21 ON076460 against the bacterial strains, *E. coli* ATCC 25922, *S. aureus* ATCC 25923, and *K. pneumonia* ATCC 13883 compared with the antibiotics, ampicillin used as a positive control. The most affected bacteria is *K. pneumoniae* ATCC 13883. **b** The antifungal activity of melanin against A. *brasiliensis, A. niger, A. sydowii, A. candidus, A. parasiticus, P. notatum, F. oxysporum*, and *P. digitatum* fungal strains compared with the antibiotics, nystatin used as a positive control. The greatest affected fungi are *P. digitatum* and *F. oxysporum*

The antibacterial activity of melanin pigment was determined against antibiotic-resistant bacterial strains in comparison with ampicillin (40 µg/mL) as a positive control. It was concluded from Fig. 6athat 100 µL of the melanin pigment displays antibacterial action against *Escherichia coli* ATCC 25922, *Staphylococcus aureus* ATCC 25923, and *Klebsiella pneumoniae* ATCC 13883 with a zone of inhibition (ZOI) of 35.16 ± 0.036, 34.27 ± 0.053, and 37.51 ± 0.012 mm, respectively. While the inhibition zones of ampicillin are 38.15 ± 0.014, 39.05 ± 0.023, and 41 ± 0.35 mm, respectively. Moreover, the results demonstrated that, the MIC values of melanin against *E. coli* and *K. pneumoniae* are 6.25 μg/mL while it registers 12.5 μg/mL on *S. aureus.* Melanin displays a dual behavior, serving as bacteriostatic at lower concentrations and bactericidal at higher ones.

According to the results, melanin seems to be more efficacious against Gram-negative than Gram-positive bacteria. Gram-negative bacteria are recognized to be more resistant to antibacterial agents because of their distinctive outer membrane and lipopolysaccharide components that distinguish them from Gram-positive bacteria [37]. Microbial diseases can be effectively managed using melanin pigment. Melanin produced from *Streptomyces* sp. represented antibacterial properties against *Lactobacillus vulgaris* and *E. coli* [38]. Also, melanin was found to have an antimicrobial effect on *Candida albicans* and *Helicobacter pylori* [39]. The melanin extracted from* Pseudomonas balearica* displayed an antimicrobial action against *S. aureus, E. coli, Candida albicans*, and the phytopathogenic bacteria *Erwinia chrysanthemi* and *E. carotovora* [40].

#### The antifungal activity

The antifungal activity of melanin extracted from the fungus *Curvularia soli* AS21 ON076460 was evaluated. The results disclose that 100 μL of melanin displays a high inhibitory effect against all tested fungi: *A. brasiliensis* AUMC 13921, *A. niger, A. sydowii, A. candidus, A. parasiticus, P. notatum, F. oxysporum,* and *P. digitatum* (Fig. 6b). The greatest affected strains are *P. digitatum* and *F*. *oxysporum* which register diameter inhibition zones of 44.25 ± 0.214 and 43.58 ± 0.158 mm, respectively. On the other hand, *A. niger* and *A. brasiliensis* are less affected with inhibition zones of 15.68 ± 0.35 and 12 ± 0.140 mm, compared to 49.58 ± 0.085, 41 ± 0.25, 20.12 ± 0.58, and 15.27 ± 0.051 mm, respectively for nystatin as a positive control.

Melanin pigment extracted from the fungus *Curvularia soli* AS21 ON076460 exhibit considered antifungal activity. A similar work has described the antifungal action of black melanin versus *A. niger, A. oryzae,* and* Penicillium* sp [41]. Moreover, melanin showed substantial antifungal efficacy toward dermatophyte fungi, *Trichophyton rubrum* and *T. simii* [12]. *Curvularia* species produce a wide variety of secondary metabolites, particularly alkaloids, polyketides, and melanin. Which have a variety of biological actions, include anti-tumer, anti-inflammatory, anti-microbial, and antioxidant [42].

**Fig. 7:**
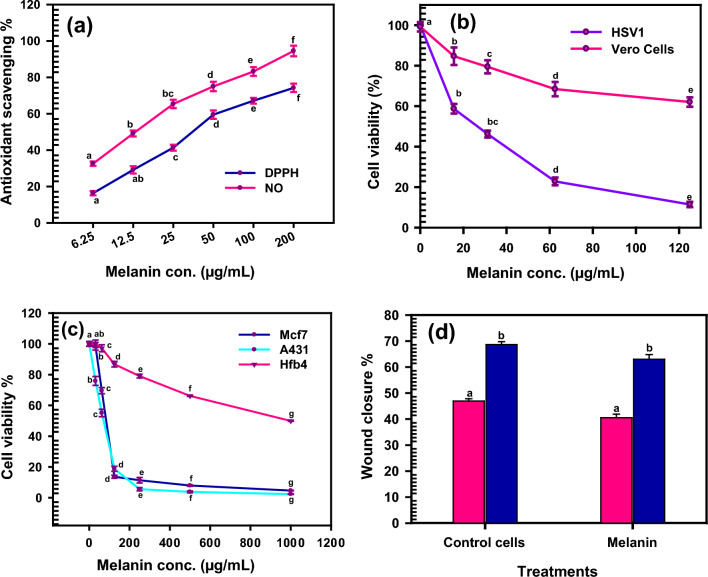
Bioassay of melanin produced by *Curvularia soli* AS21 ON076460. **a** The antioxidant activity by DPPH and NO. **b** The antiviral against HSVI and Vero cells rep* Curvularia soli* AS21 ON076460resented as cell viability (%) of viral cells. Melanin has considered cytotoxicity against the HSV1 and melanin has not cytotoxic action on normal Vero cells. (**c**) Cytotoxicity against Hfb4, McF, and A431 cells. Melanin does not apply cytotoxic effects on the tested cell lines. IC_50_ value of melanin pigment against Hfb4, Mcf7 and A431 cell lines are 350 ± 0.23, 84.23 ± 0.094 and 89.96 ± 0.16 µg/mL, and **d** wound healing activity of melanin toward human skin epithelial cells. Cell migration efficiency decreased with increased melanin concentration. The wound edges were enclosed by 63.04 ± 1.83% compared with the control (68.67 ± 1.10%) within 48 h. The results were plotted as mean values. Error bars represent the SD. According to Duncan’s multiple range test, the same letters represented not significant difference at p < 0.05

In current study, the antioxidant potency of melanin was estimated by DPPH and NO scavenging activity. The results confirm the superior antioxidant activity of melanin. The IC_50_ value for the DPPH scavenging of melanin is 42.15 ± 0.021 μg/mL, while IC_50_ for the nitric oxide scavenging is 17.0 ± 0.02 μg/mL compared to ascorbic acid as standard (Fig. 7a).

Melanin plays a crucial antioxidant role by neutralizing free radicals and limiting the production of reactive oxygen species (ROS) [43]. The functional groups of the fungal melanin structure, which offer a variety of distinctive nonequivalent binding sites for metal ions, can be used to explain the chelating ability of fungal melanin. Our findings were in accordance with those obtained by Tran-ly et al. [10], who reported that melanin had great removing free radicals activity and served as an antioxidant with useful uses in pharmaceutical medications. Both natural and synthetic melanin reveal a redox behavior, counteracting ROS as superoxide, hydroxyl radical, and singlet oxygen [44]. Interestingly, a putative natural antioxidant of fungal melanin has proven the capability to combat reactive nitrogen and oxygen species [45]. The melanin has been shown to greatly suppress the generation of lipid peroxidation and reduce the ageing process [46]. The stable free radical DPPH was effectively scavenged by melanin [40].

### The antiviral activity

The antiviral activity of melanin pigment produced by *Curvularia soli* AS21 ON076460 was assessed against HSV1, as shown in Fig. (7b) in comparison to the effect of HSV1 on Vero cells. The results indicated that melanin has no cytotoxic effect on normal Vero cells at different concentrations. On the other hand, melanin is considered cytotoxic against HSV1. MNTC and CC_50_ for melanin pigment were 125 ± 1.36 and 117.45 ± 2.02 µg/mL, respectively.

The HSV1 was found to be more sensitive to melanin produced by *Curvularia soli* AS21 ON076460. Melanin pigment registers 77% inhibition of HSV1 plaque. HSV-1 is an alpha herpes virus that infects more than 4 billion humans and the infection could be visible as herpetic keratitis, oral and genital ulcerations, encephalitis, and neonatal disease [47]. Melanin complexes have a positive effect on animal models, and they may be used as a source of biopolymers to develop new drugs that have broad therapeutic potential for infectious and viral disorders [39]. Melanin and its precursors can be used as potent antiviral agents, according to a pioneer study that demonstrated how strongly they interact with the SARSCoV2 spike protein. Melanin prevented the HIV-1 envelope glycoprotein from adhering to the MT-2 lymphoblastoid cell lines [48]. Furthermore, A unique investigation uses melanin and the enzyme CYP27B1 (regulates vitamin D production) to limit COVID-19 entrance by inactivating furin protease h, which is an essential protein in the pathogenesis [49]. Melanin reduced the replication of HIV-1 and HIV-2 in two human lymphoblastoid cell lines (MT-2 and H9) as well as phytohemagglutinin-stimulated human T cells [50].

### Cytotoxicity of melanin against normal skin cell line (Hfb4) and cancer cell lines (Mcf7 and A431)

For the potential use of fungal melanin as a cosmetic must be taken into account that it is not harmful to the skin. To estimate the probable skin irritation, these components’ cell cytotoxicity assessments must be conducted. In this study, the cytotoxic effect of *Curvularia soli* AS21 ON076460 melanin was investigated against a normal human skin cell line (Hfb4) using MTT assays, one of the best methods to investigate the cytotoxicity of many substances. The extracted melanin does not have cytotoxic effects on the tested Hfb4 at concentrations ranging from 1000 to 31.25 µg/mL (Fig. 7c). It is obvious from the obtained results that the melanin pigment of *Curvularia soli* AS21 ON076460 was safe against human skin cells with an IC_50_ value of 350 ± 0.23 µg/mL.

Next, the anticancer competence of melanin has been investigated versus two distinct malignant cell lines, Mcf7 and A431. The variation in the morphological nature of Mcf7 and A431 cell lines is the initial manifestation shown after exposure to melanin pigment. As the concentrations of melanin increased, cell viability decreased, meaning cytotoxicity increased. Cell survival rate and cytotoxicity effect are shown in Fig. 7c. Data also affirmed that the IC_50_ values of melanin pigment against Mcf7 and A431cell lines are 84.23 ± 0.094 and 89.96 ± 0.16 µg/mL, respectively.

During cancer-curing, melanin is used as anticancer therapy and to safeguard radiation patients from the adverse effects of gamma rays [12]. Furthermore, the finding publicized by Ragab et al. [51] evoked that melanin inhibited the propagation of cell lines (HEPG-2, HCT-116, and MCF-7). Melanin has an effective cytotoxic action versus different cancer cell lines (MCF7, HepG2, and HCT116) and has a minimal cytotoxic impact on normal, non-cancerous HFB4 cells suggesting that melanin can be utilized as a possible natural antitumor [3].

In vivo, the anticancer effect of melanin extracted and its arginine compounds on hepatocarcinoma-bearing mice was significantly able to reduce tumor growth [52]. Likewise, melanin has been utilized to treat a variety of tumors, disorders of the blood, immune system issues, including AIDS, and conditions caused by disruptions in cell signaling [53]. More interestingly, a study carried out by Arun et al. [12] indicated that extracellular fungal melanin was efficient against the hepatocellular cancer cell line and human epidermoid larynx carcinoma cell line (HEP-2) in a dose-dependent manner, emphasizing its promising use in chemotherapy and anticancer agents.

### In vitro wound healing influence of melanin

Within 48 h of applying melanin pigment produced from the fungus *Curvularia soli* AS21 ON076460 to human skin epithelial cells, the wound edges were enclosed by 63.04 1.83% compared to the control (68.67 1.10%). Figure 7d depicts the virtual wound closure in skin cells healed with melanin. This study indicated that when the quantity of melanin increased cell migratory efficiency was reduced.

Melanin pigment has the potential for cell migration that is crucial for wound healing. To combat slow wound healing, several melanin-doped dressings have been created in recent years [54]. Melanin and melanin-like polymeric materials have really been added to hydrogels, fibrous membranes, and coatings to enhance their physical and chemical properties [55].

Additionally, El-Naggar et al. [56] reported the anti-inflammatory performance of melanin. Melanin is widely known for having antioxidant properties that can aid in reducing the production of ROS and, in turn, help diminish inflammation. The prospective use of this pigment as an active component in tissue repair engineering has recently been the focus of research. According to several research findings, melanin thin films offer significant potential for tissue repair because they are biodegradable and have anti-inflammatory responses [57].

## Conclusion

The production of melanin pigment by black fungus *Curvularia soli* AS21 ON076460-assisted gamma rays was achieved throughout this study. Melanin pigment showed superior antibacterial action against human pathogenic bacteria (*E. coli* ATCC 25922, *S. aureus* ATCC 25923, and *K. pneumoniae* ATCC 13883) and displayed a dual behavior, serving as bacteriostatic at lower concentrations and bactericidal at higher concentrations. Moreover, melanin showed superior antifungal activity toward all tested fungi. The *Curvularia soli* AS21 ON076460 melanin pigment displayed significant antioxidant activity with IC_50_s of 42 ± 0.021 and 17 ± 0.02 µg/mL against DPPH and NO, respectively. Furthermore, the MNTC of the produced melanin was 125 µg/mL, causing 77% inhibition of the plaque of HSV1. Melanin produced the lowest apoptosis rate against a normal skin cell line (Hfb4), confirming the possibility of using it as a safe medical treatment. Moreover, melanin provoked a great apoptosis rate for human breast cancer (Mcf7) and skin cancer (A431) cell lines. Besides, the study evoked the efficacy of melanin in wound management in a human skin cell line. As a result, we recommend the possibility of benefiting from the properties of melanin in the pharmaceutical industry. The strengths of this research are that melanin is extracted from the fungus, *Curvularia soli* AS21 ON076460 in a simple way, which is a natural substance that is safe for human use at the medicinal and cosmetic levels, and biochemical analyzes show its impressive success as antimicrobials, antioxidants, and anti-inflammatory, in addition to having little cytotoxicity on normal skin cells. On the other hand, there are some points that need to be studied further, including studying the effect of pH and thermal stability of melanin, and they will be studied in the future.

## Materials and methods

### Chemicals

The chemicals and solvents employed in this study were of standard purity, brought from Sigma-Aldrich Chemical Co. (St. Louis, Missouri, 63103, USA). The fungal media were obtained from Difco (United Kingdom).

### Isolation and purification of melanin producing-fungi

Five cultivated soil samples were gathered from different localities in Egypt in clean plastic bags. Ten grams of each soil sample were mixed with 90 mL of saline and allowed to stand for 30 min on a shaker at 200 rpm. Serial dilutions (10^–1^ to 10^–4^) were performed from each sample, and then (0.1 mL) was added to potato dextrose agar (PDA) plates. All plates were incubated for 7–10 days at 28 ºC. The purification of isolated fungal colonies that exhibit a dark pigmented reverse was carried out by the agar streak method [58]. On PDA medium enriched with l-tyrosine, the acquired isolates proved to have colonies that were deep brown to black in color and were regarded as promising melanin producers. The purified isolates were sub-cultured on slants of their own isolation medium and stored at 4 ºC for further investigation in the screening program for melanin production.

### Screening of melanin production

The melanin producers’ isolates were inoculated on a PDA medium and then incubated at 28º C. For a week, the pigment formation was observed every 24 h. Melanin formation is indicated by the appearance of the black color of the dispersed pigment throughout the medium [59]. The superior melanin producer isolate was considered for molecular identification and further studies.

### Consequence of gamma irradiation doses on the melanin productivity

The gamma irradiation was accomplished at the National Centre for Radiation Research and Technology (NCRRT), Egyptian Atomic Energy Authority using ^60^Co-Gamma Cell Ge220 (Canada). For the course of the experiment, radiation was administered at a dose rate of 0.781 kGy/h under ambient circumstances. The melanin-producing fungal isolate was irradiated at 0.250, 0.5, 1.0, and 2.0 kGy. The irradiated fungal isolate at different doses was sub-cultured in 250 mL of sterilized and cooled potato dextrose broth (pH 6.0) in the dark at 28 ºC for 14 days [60].

### Identification of melanin producing fungal isolates

#### Morphological identification

The standard methods for fungal morphological characterization and species indication described by Samson et al. [61] were used. The fungal isolates were inoculated on PDA medium for 7 days. Cultural characteristics such as colony color, colony reverse pigmentation, texture, and shape were observed and documented. All isolates were also examined under a light microscope for microscopic details (Optika, Italy) [62].

#### Molecular identification

Genetic identification of the most promising melanin producer fungal isolate was performed [63]. Biospin Extraction Kits (Bioer Technology Co., Ltd., Hangzhou, P. R. China) were used to extract the DNA of the selected fungus in relation to the manufacturer’s protocol. The rDNA sequences were amplified utilizing polymerase chain reaction (PCR) upon forward and reverse primers of TCTGTAGGTGAACCTGCGG and TCCTCCGCTTATTGATATGC, respectively (synthesized by MWG-Biotech, Germany) based on conserved regions of the eukaryotic rRNA gene [64]. The amplification was achieved in a thermocycler ABI GeneAmp 9700 (Applied Biosystems, USA), which was designed for a primary denaturation cycle at 94 ºC for 5 min, then 40 cycles of denaturation for 1 min at 94 ºC.

The final sequences of a fungal isolate were revised and subjected to a Blast analysis by using the NCBI database to assign potential identity with similar sequences. Following that, the fungus isolate was allocated to its operational taxonomic unit (OTU) in accordance with measurements of sequence similarity and were then compared to other associated sequences retrieved from GenBank using ClustalX [65], BioEdit [66], and (MEGA) software ver. 6.0 [67].

#### Optimization of melanin production

P-BD analysis was used to statistically optimize the medium for the greatest melanin pigment formation and identify the variables having a positive and substantial impact on the production. The statistical software Design-Expert 7.0 (Stat Ease Inc., Minneapolis, USA) was employed (Table 1). The PB experimental design depends on the first-order model shown below, as reported by El-Batal in Eq. 3 [22]:

3 $$Y={\beta }_{0}+{\mathrm{\Sigma B}}_{{\text{i}}}{{\text{X}}}_{{\text{i}}}$$

where Y represents the response melanin, β 0 is the model intercept, B i is the linear coefficient, x i is the level of independent variable, and k is the number of involved variables.

#### Extraction of melanin pigment

After incubation, the fungal mycelia were collected by centrifugation of the fermentation broth at 11,000 rpm at 4 ºC for 15 min. The cell-free supernatant was autoclaved for 20 min in 3 mL of 1.0 M NaOH to extract the melanin pigment. To precipitate the melanin, the alkaline pigment extract was acidified to pH 2 with concentrated hydrochloric acid. The precipitate was rinsed many times with sterilized distilled water, centrifuged for 15 min at 10,000 rpm, dialyzed, and then dried at 60 ºC for 48 h. The extracted melanin pigment was dissolved in dimethyl sulfoxide (DMSO) in twice the amount of solid preparation and was preserved at −20 ºC until future use [68].

#### Solubility and stability behavior of the melanin pigment

The solubility of the melanin (50 mg/L) in water, 0.1 mol/L NaOH, 0.1 mol/L KOH, 0.2% chloroform, ethanol, and acetone was studied. Dried melanin was mixed with the solvents for 10 min in a vortex at 7,000 rpm, and then incubated for 2 hours to enable full dissolution.

To investigate the melanin decolorization, a 100 ppm of melanin solution was mixed with 1 mM hydrogen peroxide (H_2_O_2_) [69]. The precipitation of melanin with FeCl_3_ [70] and 3.0 M hydrochloric acid was determined [71]. To investigate how metal ions affect the stability of melanin, 0.5 mg of the melanin pigment was dissolved in 10 mL of a 0.1 mol/L NaOH solution that also contained 0.01 mol/L of the metal ions, Cu^2+^, Zn^2+^, Ca^2+^, Mn^2+^, and Fe^2+^. The absorbance measured at λ = 480 nm, was checked for every solution after 30, 60, and 90 min [26].

#### Tyrosinase activity assay

The tyrosinase activity of *Curvularia soli* AS21 ON076460 was evaluated by the technique designated by Zaidi et al. [72] with 50 mM sodium phosphate buffer (pH 7) as a comparator. 20 mL of Czapek’s Dox broth medium was inoculated with one loop of spores and incubated for 7 days at 28 ºC. The enzyme sample was obtained by centrifuging the culture filtrate at 7,200 rpm for 15 min. 0.3 mL of l-DOPA solution (50 MM, phosphate buffer pH 7) and 3 mL extracted enzyme were incubated for 15 min at 28º C. UV–Visible spectrophotometer was used to assess the quantity of dopachrome production at λ = 475 nm. The absorbance difference is related to the enzyme concentration. 1 unit corresponds to the catalytic transformation of 1 mol of the substrate into a byproduct in 1 min with 1.35 variations in absorbance.

#### Characterization of melanin pigment

The structural characteristic and functional groups of melanin were assessed by FT-IR spectrum measurement (JASCO FT-IR 3600 infra- red spectrometer) between 400 and 4000 cm^−1^ at a 4 cm^−1^ resolution [28]. The X-ray diffraction (XRD) analysis was performed using XRD-6000 (Shimadzu device, Japan) at wavelength 1.5418 A0, X-ray diffraction patterns Shimadzu equipment utilized Cu-K (a). In addition, the surface morphology of melanin generated was examined using a scanning electron microscope (SEM, ZEISS-EVO 15-UK). To analyze the composition of elements constituting the extracted melanin, the Energy Dispersive X-ray (EDX) was utilized.

### The bioassays of the melanin pigment

#### The antibacterial activity

The antibacterial impressions of melanin were assessed using the agar diffusion assay against three human pathogenic strains: *Escherichia coli* ATCC 25922, *Staphylococcus aureus* ATCC 25923, and *Klebsiella pneumoniae* ATCC 13883, that were kindly obtained from the Botany and Microbiology Department, Faculty of Science, Al-Azhar University (Girls Branch), Cairo, Egypt. The tested bacteria were cultured in brain heart infusion broth (BHIB) at 37 ºC and kept in saline. The bacterial inoculum (4 × 10^5^ CFU/mL) was then applied on the agar plate’s surfaces. Next, symmetrical wells with 4 mm diameter were made on the agar surface. Each well was supplied with 100 μL/well of the melanin pigment and allowed to stand for 1 h to ensure melanin diffusion. The antibiotic ampicillin (40 μg µg/mL) and distilled H_2_O were applied as positive and negative control, respectively. After 24 h of incubation at 35º C, the inhibitory zones were recorded [73].

The minimal inhibitory concentration (MIC) of melanin was determined in accordance with Fathy et al. [23]. *E. coli* ATCC 25922, *S. aureus* ATCC 25923, and *K. pneumoniae* ATCC 13883 colonies were cultured individually for 18 h in a nutrient broth medium at 30 ºC. A series of dilutions were subsequently performed to produce a final inoculum containing approximately 10^4^ CFU/mL. Bacterial suspensions were administered at concentrations ranging from 3.25 to 200 µg/mL at the surface of nutrient agar plates that had been provided with melanin. The experiment was carried out in triplicate, and all plates were incubated overnight at 37º C. The MICs were determined to be the lowest concentrations at which no bacterial growth was visible on the agar plates.

#### The antifungal activity

The fungal strains *Aspergillus niger, Aspergillus brasiliensis* AUMC 13921*, Aspergillus sydowii, Aspergillus candidus, Aspergillus parasiticus, Penicillium notatum, Fusarium oxysporum,* and* Penicillium digitatum* were obtained from the Botany and Microbiology Department, Faculty of Science, Al-Azhar University (Girls Branch), Cairo, Egypt. The antifungal efficacy of melanin on the tested fungi was assessed by the agar well method [74]. The sterilized PDA medium was inoculated with the tested fungi separately. Wells (6 mm) were loaded with 100 µL of melanin (200 μg/mL). Nystatin (NS) was used as the standard antifungal positive control. The antifungal activity was determined in triplicate. The cultures were incubated at 28º C for 5 days. The inhibitory action of melanin was deliberate in terms of inhibition zone diameter (mm).

#### The antioxidant activity

The antioxidant activities of melanin were investigated using DPPH [75] and nitric oxide (NO) [76] scavenging activity. The following formula (Eq. 4) was used to compute the % radical scavenging activity [75]:

4 $$\mathrm{Scavenging\, activity\, \% }=\left(1-\left[\frac{{A}_{t}}{{A}_{c}}\right]\right)\times 100$$

where A_c_ was the absorbance of DPPH and NO while A_t_ was the absorbance of melanin and ascorbic acid.

#### The antiviral activity of melanin using (MTT assay protocol)

The cytotoxicity of melanin on Vero cell and Herpes simplex virus (HSB1) at concentrations of 31.25, 62.5, 125, 250, 500, and 1000 µg/mL was measured [77]. The optical densities were recorded at λ = 560 nm. Then the CC_50_ and IC_50_ were determined. The maximum non-toxic concentration (MNTC) of the melanin pigment was detected. The 50% cytotoxic concentration (CC_50_) is the concentration of melanin causing 50% reduction of the cell viability related to the control.

#### Cytotoxicity test versus normal skin cell line (Hfb4) and cancer cell lines (Mcf7 and A431)

The cytotoxic action of melanin on the normal skin Hfb4 cell line was detected, according to Algotiml et al. [78]. A volume of 100 µL/well of two fold dilutions of the melanin pigment at concentrations of 31.25, 62.5, 125, 250, 500, and 1000 µg/mL was injected into the 96-well microliter plate containing 1 × 10^5^ normal skin cells (Hfb4). For MTT metabolization, the samples were incubated for 4 h at 37º C, 5% CO_2_. Three wells were left as controls and do not include melanin. The absorbance at λ ꞊ 570 nm was measured using a plate reader. The calculation of cell viability in the GraphPad Instate software version 3.1 was used to determine the IC_50_ (the concentration of the melanin that resulted in 50% of cell death).

The cytotoxic effect of the melanin pigment has been investigated in a human breast cancer (Mcf7) and a model human cell line (epidermoid carcinoma) (A431) using the MTT assay [78]. The anti-tumor efficacy of the melanin extract was investigated using a phase-contrast inverted microscope (Olympus, Japan). The IC_50_ values were determined with the aid of the GraphPad Instate program.

#### In vitro assessment of wound healing activity of melanin by a scratch assay method

To estimate the wound healing capacity of melanin, the skin epithelial cell line was obtained from the Cancer Institute in Cairo, Egypt. The cells were grown to confluence (complete confluence to develop a complete monolayer sheet) in six multi-well plates [79]. Straight scratching was carried out with a sterilized yellow pipette edge, and the cell line was subsequently subjected to100 µL of melanin (200 µg/mL) for a period of 48 h. Photographs of cell migration were obtained throughout the healing process, from wound inception to wound closure. Afterward, using the 10 × objective, imaging of each wound site was executed. Finally, the results were expressed as mean ± SD. *P* ≤ 0.05 was regarded as a statistical significance.

### Statistical analysis

All analyses were done using ANOVA at p ≤ 0.05 and labeled as mean ± SD. Duncan’s multiple range test was applied to compare the treatments with significantly different means using distinct superscripts (p ≤ 0.05). IBM Corp analyzed the information (Released 2016. IBM SPSS Statistics is Version 24.0. Armonk, NY: IBM Corp). Sigma Plot Version 12 (Spw.exe) was used to plot the results. Additional file 1: Scheme S1 summarized the materials and methods section.

### Supplementary Information

**Additional file1: Table S1.** Morphological examination of the melanin producing strains. **Table S2.** Response of melanin pigment produced by *Curvularia soli* AS21 ON076460 irradiated at 1.0 kGy; Analysis of variance (ANOVA) for selected factorial model. **Table S3.** Response of melanin pigment produced by *Curvularia soli* AS21 ON076460 (Un-irradiated control); Analysis of variance (ANOVA) for selected factorial model. **Table S4.** Physical and chemical characteristics of melanin produced by *Curvularia soli* AS21 ON076460. **Scheme S1.** The flowchart summarized the materials and methods section.

## Data Availability

The data that support the findings of this study are available from the corresponding author upon reasonable request.

## Abbreviations

A431: Skin cancer cell lines
AIDS: Acquired immunodeficiency syndrome
BHIB: Brain heart infusion broth
CzA: Czapek’s agar medium
C: Carbon
CFU/mL: The colony forming unit/milliliter
CC: Cytotoxic concentration
CLSI: The Clinical & Laboratory Standards Institute
DHN: 1,8-Dihydroxy naphthalene
DPPH: 2,2-Diphenyl-1-Picrylhydrazyl
GDHB: Glutaminyl-3,4-Dihydroxybenzene
FT-IR: Fourier transforms infrared spectroscopy
HSV: Herbs simplex virus
h.: Hour
HIV: Human immune-deficiency viruses
HFB4: Human normal fibroblast cells
HEPG-2: Human liver cancer cell line
MCF-7: Breast cancer cell line
HCT-116: Human colorectal carcinoma cell line
LSD: Least significant difference
kGy: Kilo gamma rays
MNTC: Maximum non-toxic concentration
MIC: Minimum inhibitory concentration
ML: Machine learning
NO: Nitric oxide
NJ: Neighbor-joining
N: Nitrogen
NS: Nystatin
NCBI: National center for technological information
NCRRT: National center for radiation research and technology
OECTs: Organic electrochemical transistors
OTU: Operational taxonomic unit
PDA: Potato dextrose agar
PBD: Potato dextrose broth
