# Supplementary material for: Production and characterization of melanin pigment from black fungus Curvularia soli AS21 ON076460 assisted gamma rays for promising medical uses
Source: Microb Cell Fact. 2024 Feb 26;23:68. doi: 10.1186/s12934-024-02335-y (PMC10895916; doi:10.1186/s12934-024-02335-y)
Supplement: Supplementary file 1 — Additional file1: Table S1. Morphological examination of the melanin producing strains. Table S2. Response of melanin pigment produced by Curvularia soli AS21 ON076460 irradiated at 1.0 kGy; Analysis of variance (ANOVA) for selected factorial model. Table S3. Response of melanin pigment produced by Curvularia soli AS21 ON076460 (Un-irradiated control); Analysis of variance (ANOVA) for selected factorial model. Table S4. Physical and chemical characteristics of melanin produced by Curvularia soli AS21 ON076460. Scheme S1. The flowchart summarized the materials and methods section. [file 12934_2024_2335_MOESM1_ESM.docx]

**Scheme 1. The flowchart summarized the materials and methods section**

**Table 1. Morphological examination of the melanin producing strains**

| Isolates | Identification | Culture | Microscopic examination |
| --- | --- | --- | --- |
| F1 | ***Curvularia* sp.** | 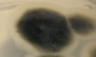 | 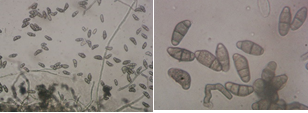 |
| F2 | ***Bipolaris* sp.** | 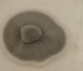 | 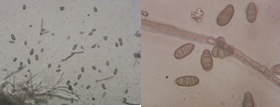 |
| F3 | ***Alternaria* sp.** | 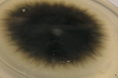 | 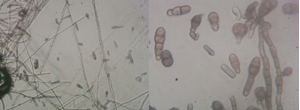 |
| F4 | ***Alternaria* sp.** | 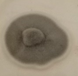 | 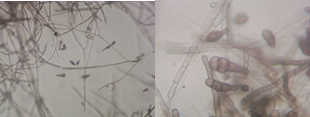 |
| F5 | ***Alternaria* sp.** | 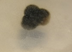 | 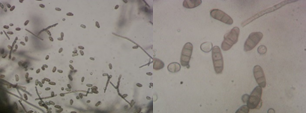 |
| F6 | ***Curvularia* sp.** | 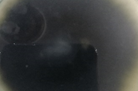 | 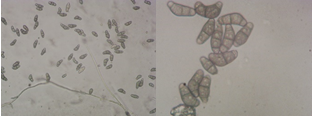 |
| F7 | ***Cladosporium* sp.** | 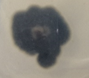 | 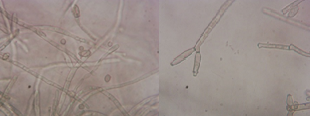 |
| F8 | **unidentified** | 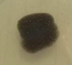 | 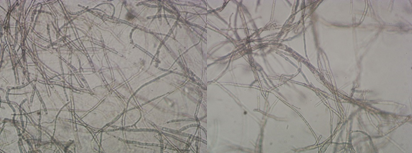 |
| F9 | **unidentified** | 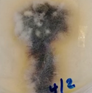 | 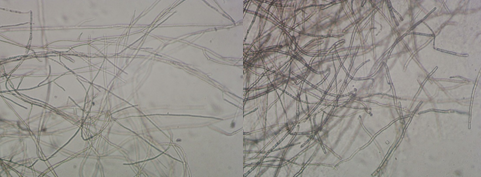 |
| F10 | ***Curvularia* sp.** | 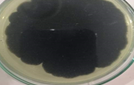 | 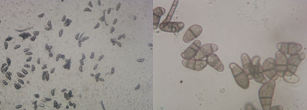 |

**Table 2. Response of melanin pigment produced by *Curvularia soli* AS21** **ON076460 irradiated at 1.0 kGy; analysis of variance (ANOVA) for selected factorial model**

| **Source** | **Sum of square** | **df** | **Mean square** | **F value** | **p-value**  **prob>F** |
| --- | --- | --- | --- | --- | --- |
| **Model** | 3.53 | 11 | 0.32 | - | - |
| **A- Heavy metal** | 0.20 | 1 | 0.20 |  |  |
| **B- C.source** | 0.69 | 1 | 0.69 |  |  |
| **C- N.source** | 0.18 | 1 | 0.18 |  |  |
| **D-Temperature** | 0.70 | 1 | 0.70 |  |  |
| **E-Ph** | 0.46 | 1 | 0.46 |  |  |
| **F- Time** | 0.015 | 1 | 0.015 |  |  |
| **G-Lumination** | 0.65 | 1 | 0.65 |  |  |
| **H-Shaking** | 0.34 | 1 | 0.34 |  |  |
| **J-Medium type** | 0.028 | 1 | 0.028 |  |  |
| **K-Salinity** | 1.045E-003 | 1 | 1.045E-003 |  |  |
| **L-L.tyrosine** | 0.25 | 1 | 0.25 |  |  |
| **Residual** | 2.56 | 11 | 0.23 |  |  |
| **Pure error** | 0.000 | 0 |  |  |  |
| **Cor total** | 3.53 | 11 |  |  |  |

**Table 3. Response of melanin pigment produced by *Curvularia soli* AS21 ON076460 (Un-irradiated control); Analysis of variance (ANOVA) for selected factorial model**

| Source | Sum of square | Df | Mean square | F-value | p-value  prob>F |
| --- | --- | --- | --- | --- | --- |
| Model | 0.000 | 0 | - | - | - |
| Residual | 2.56 | 11 | 0.23 |  |  |
| Control | 2.56 | 11 | - |  |  |
| Std. Dev. | 0.48 | R. square | 0.0000 |  |  |
| Mean | 2.50 | Adj R^2^ | 0.0000 |  |  |
| C.V.% | 19.32 | Pred R^2^ | -0.1901 |  |  |
| Press | 3.05 | Adeq. Precision |  |  |  |

**Table 4. Physical and chemical characteristics of melanin produced by *Curvularia soli* AS21 ON076460**

| Test | Result |
| --- | --- |
| Color | Black |
| Solubility in water | - |
| Solubility in NaOH | + |
| Solubility in KOH | + |
| Solubility in chloroform | - |
| Solubility in ethanol | - |
| Solubility acetone | - |
| Decolorization by oxidants agents (H_2_O_2_) | + |
| Precipitation with HCl | + |
| Precipitation for polyphenols with FeCl_3_ | + |

(+), (-) are the positive and negative responses
